# Quality of Life in Patients with Inflammatory Bowel Diseases Is Associated with Affective Temperament Traits: A Cross-Sectional Survey of a Polish Clinical Sample

**DOI:** 10.3390/jcm14031018

**Published:** 2025-02-05

**Authors:** Anna Mokrowiecka, Magdalena Kopczynska, Alina Borkowska, Ewa Malecka-Wojciesko

**Affiliations:** 1 Department of Digestive Tract Diseases, Medical University of Lodz, 90-419 Lodz, Poland; 2 Clinical Neuropsychology Unit, Collegium Medicum Bydgoszcz, 85-094 Bydgoszcz, Poland; alab@cm.umk.pl

**Keywords:** temperament, inflammatory bowel disease, quality of life

## Abstract

**Background:** Affective temperaments can be considered the subclinical manifestations of affective and stress-related disorders, which could have a relationship with many chronic diseases. The purpose of this study was to explore the influence of affective temperament traits on disease-specific quality of life in patients with ulcerative colitis (UC) and Crohn’s disease (CD), two types of inflammatory bowel disease (IBD). **Methods:** The patients completed the Temperament Evaluation of the Memphis, Pisa, Paris, San Diego-Auto-questionnaire (TEMPS-A), which is the 110-item self-reported assessment for five dimensions of temperament: depressive, cyclothymic, hyperthymic, irritable, and anxious, already validated in Poland. For comprehensive assessment of the health-related quality of life (HRQoL), the Inflammatory Bowel Disease Questionnaire (IBDQ) was applied. **Results:** The study included 116 patients with IBD-61 with UC and 55 with CD, with mean age 43 years, in remission, without serious mental or medical co-morbidities. Mean HRQoL in patients with IBD was poor and mean IBDQ scores were 145, despite clinical remission. A significant negative correlation was found between HRQoL in all the IBDQ domains and TEMPS-A traits: D (*p* < 0.001), C (*p* < 0.01), I (*p* < 0.05), and A (*p* < 0.001). No significant correlation between hyperthymic temperament and IBDQ scores was found. **Conclusions:** Poor quality of life in IBD could be associated with affective temperament. Affective temperament traits should be taken into account when identifying patients at risk of worse IBD course and further introducing personalized therapy.

## 1. Introduction

Inflammatory bowel diseases (IBD), including ulcerative colitis (UC) and Crohn’s disease (CD), are chronic remitting-relapsing diseases of the gastrointestinal tract. The prevalence of IBD today exceeds 0.3% of the total population in countries like Canada, the United States, New Zealand, Denmark, Germany, and the United Kingdom [1]. Patients with IBD deal with many problems, such as stress related to their health issues, fear of relapses, emotional and cognitive adaptation to the disease, psychological effects of illness and treatment, and changes in the family organization and the social environment [2,3,4].

Health-related quality of life (HRQoL) is poor in IBD compared to other chronic conditions like rheumatoid arthritis, asthma, and migraine headaches [5]. In many studies, this low quality of life has been correlated with disease activity [2,6,7,8,9]. However, the reduced quality of life may not always be explained by the disease exacerbation, and the level of HRQoL does not always depend on the severity of the disease symptoms. There are many patients who have psychological difficulties despite being in remission [10,11,12]. In patients with IBD, compared to the general population, depression and anxiety disorders and symptoms are more commonly reported [13]. Epidemiological studies show that anxiety and depression are the most prevalent psychiatric disorders among patients with IBD [14,15,16,17].

Temperament is regarded as an inherited part of personality and represents the biologically stable core of emotional reactivity [18]. Affective temperaments (depressive, cyclothymic, hyperthymic, irritable, and anxious) are subclinical, trait-related manifestations and commonly the antecedents of minor and major mood disorders [19]. In the literature, affective temperaments have been reported to correlate with a broad variety of pathological conditions ranging from somatic to psychiatric disorders [20]. Besides their associations with affective disorders, substance abuse, and eating disorders, affective temperaments have been found to be related to somatic diseases, like hypertension, coronary events, obesity, and diabetes mellitus [20,21,22,23,24,25]. Data on the influence of affective temperaments on gastrointestinal disturbances are sparse [26]. Moreover, psychological studies rarely present data from Eastern Europe.

Our study aimed at assessing whether affective temperament traits could influence HRQoL in patients with IBD. Identifying the variables associated with the decline in HRQoL could be important in order to identify patients at risk of poorer biopsychosocial outcomes and further introducing personalized therapy.

## 2. Methods

Participants completed the Temperament Evaluation of the Memphis, Pisa, Paris, San Diego-Auto-questionnaire (TEMPS-A), which is the 110-item self-related assessment tool for evaluation of five dimensions of affective temperament: depressive (D), cyclothymic (C), hyperthymic (H), irritable (I), and anxious (A), already validated in Poland [27,28]. The scoring for each scale was calculated using a mathematical formula: sum of scoring of the variables belonging to each category/number of variables (nv): (*v*1 + *v*2 + ….*vx*)/nv. The following items belonged to such categories: depressive temperament, items 1–21 (21 traits); cyclothymic temperament, items 22–42 (21 traits); hyperthymic temperament, items 43–63 (21 traits); irritable temperament, items 64–84 (21 traits); anxious temperament, items 85–110 (26 traits). Subjects were asked to answer for all 110 questions YES or NO, where yes was marked as “1” and no was marked as “0”. A higher score indicated higher intensity of affective temperament in the given category.

Affective temperaments are not associated with poorer overall outcomes when they appear in a mild form, but when they occur in extreme form [20]. Thus, we categorized patients into groups of low intensity of a given temperament (<0.5), moderate (0.5–0.7), and strong intensity of this temperament (>0.7).

To obtain comprehensive assessment of HRQoL, patients completed the Inflammatory Bowel Disease Questionnaire (IBDQ). The IBDQ is a 32-item questionnaire measuring quality of life in IBD patients. Each item is scored on a 7-level Lickert’s scale (1–7), where 1 means the lowest and 7 the best quality of life of investigated patients. The IBDQ consists of four subscales, measuring different aspects of quality of life: emotional function (worried, feeling depressed, frustrated), social function (function in leisure and work), systemic symptoms (being tired, having sleep problems, lack of energy), and bowel function (stool consistency, frequency, pain) [29]. Remission was obtained with cutoff values of 168 [29]. Sociodemographic variables as well as clinical features were also collected.

### 2.1. Statistical Analysis

The distribution of each variable was checked using the Shapiro–Wilk test. Homogeneity of variance was evaluated using the Levene test. The criteria for normality were not met, therefore non-parametric tests were used. Two groups were compared using a Mann–Whitney U-test, and more than two groups were compared using the Kruskal–Wallis analysis of variance. A *p* value of <0.05 was considered the measure of statistical significance.

### 2.2. Ethical Statement

Permission for this study was obtained from the Ethics Committee of Medical University of Lodz (protocol code RNN/78/12/KE 17 April 2012).

The purpose and procedure of this study was explained to the patients, who expressed interest in participation. In line with the Bioethics Committee’s consent guidelines, patients were asked to give their written informed consent to participate in this study. This study was conducted in accordance with the Declaration of Helsinki.

## 3. Results

Overall, 116 patients with IBD were recruited to this cross-sectional survey study from the gastroenterology university clinic in Lodz, Poland. Patients were eligible for the study if they had a diagnosis of UC or CD verified by endoscopy and histology, were >18 years old, were on stable medication (4 weeks), and were willing to participate in a study. Exclusion criteria were active IBD phase, severe mental disorders, current psychotherapy, and accompanying major medical illness.

All patients with IBD were in clinical and endoscopic remission and presented Crohn’s Disease Activity Index (CDAI) scores less than 150 for patients with CD and Mayo scores less than three for patients with UC. Basic demographic and clinical characteristics of patients with IBD are shown in Table 1.

Mean HRQoL in patients with IBD was moderate and mean IBDQ scores were 145 (range 61–220). Among patients with IBD, those with UC had lower IBDQ scores compared to patients with CD in all domains, but the difference was not statistically significant (Table 2).

The most common combination of affective traits was: depressive, cyclothymic, and anxious and these traits reached the highest scores (Table 3). Irritable temperament scores were significantly higher in patients with CD compared to those with UC (*p* < 0.01). Other TEMPS-A mean scores of patients with CD did not differ as compared to patients with UC (See Appendix A).

When categorized into a group that had a low intensity of a given temperament (<0.5), moderate (0.5–0.7), and a strong intensity of this temperament (>0.7), in 25 patients (22%) we found three and more affective temperament traits with scores of more than 0.5. In 37 patients (33%), at least one score was more than 0.7.

A significant negative correlation was found between HRQoL in all IBDQ domains and TEMPS-A traits: D (*p* < 0,001), C (*p* < 0,01), I (*p* < 0,05), and A (*p* < 0,001) (Table 4). No significant correlation between hyperthymic temperament and IBDQ scores was found (Table 4).

TEMPS-A correlation with clinical factors analysis was also carried out for the effects assessment of gender, age, BMI, and history of pharmacotherapy and surgical treatment (data shown in Appendix A). We only found lower cyclothymic TEMPS scores in the group of patients treated with anti-TNF medications, lower depressive scores in patients treated with AZT, and more women with higher intensity anxious TEMPS traits (Appendix A).

## 4. Discussion

Rubin et al. [5] confirmed that patients with UC experience a significantly higher psychological burden, and their symptoms are frequently more disruptive to their lives, relationships, and emotional state than for patients with rheumatoid arthritis, asthma, or migraine headaches. There is growing interest in identifying factors involved in HRQoL decrease in patients with IBD [30,31]. In several studies authors point to the disease activity’s impact on the wellbeing in this group [32,33]. However, among patients with IBD there is a considerable number of individuals who have low QoL despite being in clinical remission [30]. The literature shows that they report worries and concerns regarding complications, stigmatization, or intimacy, as well as concerns regarding their management plan or effective treatments—worries which are present also during remission [5,34].

Our patients’ quality of life, despite clinical and endoscopic remission, was poor. Using the TEMPS-A questionnaire we found that there is a considerable number of patients with IBD with high intensity of affective personality traits. Furthermore, we found a significant negative correlation between HRQoL in the IBDQ domains and TEMPS-A traits: depressive, cyclothymic, irritable, and anxious. The findings indicate that the greater the share of affective traits, the poorer the quality of life in all but one of the IBDQ domains—emotional, social, systemic, and bowel.

The hyperthymic temperament, which was not a significant correlate of HRQoL in our study, has been reported to have a uniquely protective effect against mental disorders [35]. Persons with this type of temperament have chronic low-grade hypomanic symptoms—high energy, the need for less sleep than others, chronic optimism, and risk taking. Hyperthymic temperament is independent from other affective temperaments and this difference is well seen in our study. In Bieliński et al. [26], hyperthymic temperament in patients with IBD was associated with lower symptoms of anxiety and depression measured with the Hospital Anxiety and Depression Scale.

On the other hand, other dimensions of affective temperament in Bieliński et al. [26] exhibited statistically significant positive correlations with the severity of anxiety and depression, except for the irritable temperament, which did not reach significance in patients with CD. There is a big difference between the hyperthymic temperament and the cyclothymic, depressive, irritable, and anxious ones, which are closer to mental and substance use disorders, and could determine somatic diseases [20]. These characteristic differences between hyperthymic and other affective traits were also seen in our study.

In our study the results of the TEMPS-A questionnaire did not correlate with factors that could worsen the course of the disease, such as the number of relapses, duration of the disease, and the history of at least one surgical operation. Similarly, in the study by Bieliński et al. [26], the number of surgical procedures was not related to the TEMPS-A subscales for patients with IBD. The correlation of the TEMPS-A scale with the total score of the CDAI clinical scale did not show significant results [26].

To date, the temperamental profile’s association with HRQoL has not been examined in a sample of outpatients with IBD. A somewhat similar study was conducted by Vidal et al. [30], who used the Temperament and Character Inventory for assessing personality traits and the Hospital Anxiety and Depression Scale for assessing psychopathology and its influence on HRQoL in patients with IBD. These authors showed that disease activity and psychological distress were the strongest predictors of QoL impairment [30]. Poor HRQoL concerned the patients who were more insecure (high scores on Harm Avoidance), obsessive (high scores on Persistence), and less able to deal with problems (low scores on Self-Directness). Only one personality trait (Self-Directness) was an independent predictor of one QoL dimension (Emotional dimension). The authors concluded that personality may be an indirect predictor of psychological distress, which directly impacts on QoL [30].

Moreno-Jiménez B. et al. [36] examined 120 patients with IBD and concluded that some personality factors are associated with poorer QoL of these patients. The authors used the IBDQ, the Rosenberg Self-Esteem Scale, and the Neuroticism scale of the Eysenck Personality Inventory. They found self-esteem was the factor most closely related to social functioning, and neuroticism was the most closely related to the four indicators of quality of life [36].

Affective temperament is considered a stable construct associated with genetic transmission and could serve as a phenotype to detect genes responsible for a susceptibility to affective disorders [28,37]. However, some authors have suggested that there are factors which could impact temperamental dimensions, like the immune system or chronic inflammation. Pessimism contributed to an increase of inflammation markers, like IL-6 and the C reactive protein [38]. It has been shown that a positive attitude may affect the immune system, by for example lowering the IL-6 response to the stress factors [39,40]. Patients suffering from other immune-mediated inflammatory diseases, such as multiple sclerosis or rheumatoid arthritis, have been shown to be at increased risk of psychiatric comorbidity [41]. There were associations between adolescent temperament and inflammation. Higher negative emotionality was significantly associated with higher CRP levels. Furthermore, these associations were larger than those for depressive symptoms [42].

The question remains as to whether inflammation can affect temperament, or if the mechanism is opposite. This issue could explain different disease courses in patients with IBD. Stress is one of the strongest predictors of disease course in IBD, in which the immune system responds in an exaggerated way to gut bacteria, a reaction triggered by environmental factors [43]. IBD is increasingly considered to be a disorder of the gut–brain axis. Patients with anxiety whose IBD was in remission at baseline had a twofold increase in the risk of a future flare of IBD. The inflammatory changes in IBD can lead to sensitization, even when patients are in remission [43]. It could also explain poor quality of life in our patients with IBD in remission.

On the other hand, inflammatory mechanisms might have a role in the etiology of depression, with plasma levels of pro-inflammatory cytokines such as TNF, IL-1, and IL-6 predicting its onset [43,44]. Antidepressants are hypothesized to treat mental illness via immunoregulatory pathways, by decreasing levels of pro-inflammatory cytokines such as TNF52, IL-6, IL-1β, and IL-10 [43,45]. More research on psychoneuroimmunology or the influence of environmental factors on the affective temperament is needed.

### Limitations and Strengths of Current Report

To our knowledge, this is the first study evaluating the affective temperament using the TEMPS-A questionnaire in patients with IBD in comparison to the quality of life of these patients. Our findings show a strong association between four affective temperament traits and worse quality of life results. We have noticed a protective effect of hyperthymic temperament on quality of life. TEMPS-A is a simple tool for assessing vulnerability to psychiatric disorders and prognostic evaluation in IBD patients.

The main limitations of our study are the relatively small sample of patients and the cross-sectional type of the study, which does not allow the generalization of the findings. Another disadvantage of our study is the lack of laboratory tests, including inflammatory factors, and their correlation with TEMPS-A test results. Those tests with genetic polymorphisms and changes in serotoninergic neurotransmission will enable the creation of subgroups of patients with IBD for administration of individualized and better adjusted treatment strategies against mood disorders.

## 5. Conclusions

Our study demonstrated that affective temperament traits have a negative impact on quality of life in patients with IBD. There is a considerable number of individuals who have low QoL despite being in clinical remission, and these findings will promote a better understanding of these patients. Personality traits should be taken into account when using IBDQ in studies as well as in management with patients with IBD.

Affective temperaments represent adaptive dispositions whose dysregulation can lead to full-blown affective pathology. We have identified a subgroup of patients with IBD with higher than 0.7 intensity of affective traits and we think that these patients need special medical attention.

Controlling and minimizing the symptoms of the disease, along with the identification and treatment of psychopathology, should become integral aspects of IBD care to improve HRQoL of these patients. These results provide additional evidence for the need for personalized interventions in this group of patients.

## Figures and Tables

**Table 1:** Basic demographic and clinical characteristics of patients with IBD.

		CD (*n* = 55)	UC (*n* = 61)	IBD (*n* = 116)
**Mean Age, Range (Years)**		39 (19–69)	46 (21–84)	43 (19–84)
Male gender (*n* (%))		24 (44%)	31 (51%)	55 (47%)
Mean BMI (kg/m^2^)		23 (16–34)	24.4 (14–37)	23.87 (14–37)
Ongoing pharmacotherapy *n* (%)	5-amino- ASA	44 (80)	59 (97)	103 (89)
	Azathiopryne	26 (47)	10 (16)	36 (31)
	Anty-TNF	31 (56)	4 (6)	35 (30)
Medical history *n* (%)	Relapses			
	0	6 (11)	3 (5)	10 (7)
	1×	11 (20)	10 (16)	21 (18)
	2–5×	27 (49)	32 (52)	58 (50)
	>5×	11 (20)	16 (26)	27 (23)
	Surgery *	23 (42)	7 (11)	30 (26)
	Disease duration			
	<1 year	3 (5)	1 (2)	4 (3)
	<10 years	43 (78)	43 (70)	86 (74)
	10–20 years	8 (14)	8 (13)	16 (14)
	>20 years	1 (2)	9 (15)	10 (9)

* Number of patients who had undergone at least one surgery.

**Table 2:** IBDQ mean scores in IBD groups.

Mean Scores IBDQ	IBD	CD	UC	Statistical Significance
IBDQ bowel	48.86	50	47.5	*p* > 0.05
IBDQ emotion	53	54	52.5	*p* > 0.05
IBDQ systemic	19.9	20.5	19.4	*p* > 0.05
IBDQ social	23.6	24	23	*p* > 0.05
IBDQ total	145	148.6	142.5	*p* > 0.05

**Table 3:** Temperament Evaluation of the Memphis, Pisa, Paris, San Diego-Auto-questionnaire (TEMPS-A) results in patients with IBD.

	Mean Scores	Median	Minimum	Maximum	SD
TEMPS-D	0.39	0.33	0.05	0.95	0.18
TEMPS-C	0.42	0.38	0.00	1.00	0.25
TEMPS-H	0.41	0.38	0.05	0.95	0.20
TEMPS-I	0.23	0.22	0.00	0.80	0.18
TEMPS-A	0.387155	0.346154	0.00	1.00	0.24

**Table 4:** ANOVA statistics correlation between IBDQ domains and TEMPS-A scores *.

	TEMPS-A Anxious		TEMPS-A Irritable		TEMPS-A Cyclothymic		TEMPS-A Depressive		TEMPS-A Hyperthymic	
	F	Sig.	F	Sig.	F	Sig.	F	Sig.	F	Sig.
**bowel**	6.570	**0.012**	6.560	**0.012**	3.971	**0.049**	6.253	**0.014**	0.094	0.759
**emotional**	23.878	**0.000**	4.669	**0.033**	13.213	**0.000**	21.579	**0.000**	0.865	0.354
**systemic**	13.039	**0.000**	4.071	**0.046**	6.974	**0.010**	18.185	**0.000**	1.127	0.291
**social**	6.808	**0.010**	2.105	0.150	4.834	**0.030**	8.601	**0.004**	0.476	0.492
**IBDQ**	15.523	**0.000**	5.489	**0.021**	9.094	**0.003**	16.258	**0.000**	0.368	0.546

* The mean difference is significant at the 0.05 level (bold).

## Data Availability

No new data were created or analyzed in this study. Data sharing is not applicable to this article.

## Appendix A

**Table A1:** Temperament Evaluation of the Memphis, Pisa, Paris, San Diego-Auto-questionnaire (TEMPS-A) results correlation with factors of clinical course of IBD.

		TEMPS-D				Total		p
		<0.5		≥0.5				
		N	%	N	%	N	%	
group	CD	40	50	12	40	52	47.3	0.3495
	UC	40	50	18	60	58	52.7	
Gender	0	40	50	17	56.7	57	51.8	0.5331
	1	40	50	13	43.3	53	48.2	
ASA	0	9	11.3	2	6.7	11	10	0.4755
	1	71	88.8	28	93.3	99	90	
AZT	0	51	63.8	26	86.7	77	70	0.0195
	1	29	36.3	4	13.3	33	30	
Anty-TNF	0	53	66.3	25	83.3	78	70.9	0.0789
	1	27	33.8	5	16.7	32	29.1	
surgery	0	59	73.8	23	76.7	82	74.5	0.7545
	1	21	26.3	7	23.3	28	25.5	
		**TEMPS-C**						***p***
		**<0.5**		**≥0.5**				
		**N**	**%**	**N**	**%**			
group	CD	30	44.1	22	52.4			0.399
	UC	38	55.9	20	47.6			
gender	0	33	48.5	24	57.1			0.3797
	1	35	51.5	18	42.9			
5-ASA	0	5	7.4	6	14.3			0.239
	1	63	92.6	36	85.7			
AZT	0	46	67.6	31	73.8			0.4932
	1	22	32.4	11	26.2			
Anty-TNF	0	53	77.9	25	59.5			0.0388
	1	15	22.1	17	40.5			
surgery	0	52	76.5	30	71.4			0.5553
	1	16	23.5	12	28.6			
		**TEMPS–H**						***p***
		**<0.5**		**≥0.5**				
		**N**	**%**	**N**	**%**			
group	CD	31	44.9	21	51.2			0.5227
	UC	38	55.1	20	48.8			
gender	0	36	52.2	21	51.2			0.9228
	1	33	47.8	20	48.8			
5-ASA	0	8	11.6	3	7.3			0.4697
	1	61	88.4	38	92.7			
AZT	0	52	75.4	25	61			0.1114
	1	17	24.6	16	39			
Anty-TNF	0	53	76.8	25	61			0.077
	1	16	23.2	16	39			
surgery	0	51	73.9	31	75.6			0.8434
	1	18	26.1	10	24.4			
		**TEMPS–I**						***p***
		**<0.5**		**≥0.5**				
		**N**	**%**	**N**	**%**			
group	CD	44	43.6	8	88.9			**0.0091**
	UC	57	56.4	1	11.1			
gender	0	53	52.5	4	44.4			0.6441
5-ASA	0	9	8.9	2	22.2			0.2021
	1	92	91.1	7	77.8			
AZT	0	71	70.3	6	66.7			0.8199
	1	30	29.7	3	33.3			
Anty-TNF	0	73	72.3	5	55.6			0.2899
	1	28	27.7	4	44.4			
surgery	0	76	75.2	6	66.7			0.5712
	1	25	24.8	3	33.3			
		**TEMPS–A**						***p***
		**<0.5**		**>=0.5**				
		**N**	**%**	**N**	**%**			
group	CD	34	46.6	18	48.6			0.837
	UC	39	53.4	19	51.4			
gender	0	32	43.8	25	67.6			**0.0186**
	1	41	56.2	12	32.4			
5-ASA	0	7	9.6	4	10.8			0.8401
	1	66	90.4	33	89.2			
AZT	0	50	68.5	27	73			0.6281
	1	23	31.5	10	27			
Anty-TNF	0	53	72.6	25	67.6			0.5828
	1	20	27.4	12	32.4			
surgery	0	54	74	28	75.7			0.8464
	1	19	26	9	24.3			

**Table A2:** Temperament Evaluation of the Memphis, Pisa, Paris, San Diego-Auto-questionnaire (TEMPS-A) results correlation with age and BMI of patients with IBD.

	TEMPS-D			
	**<0.5**		**0.5–0.7**	
	**Mean**	**Standard Deviation**	**Mean**	**Standard Deviation**
age	42.81	16.45	44.3	13.48
BMI	23.83	4.6	23.87	4.38
	**TEMPS-C**			
	**<0.5**		**0.5–0.7**	
	**Mean**	**Standard Deviation**	**Mean**	**Standard Deviation**
age	43.73	16.31	42.39	14.63
BMI	24.18	4.77	23.29	4.09
	**TEMPS-H**			
	**<0.5**		**0.5–0.7**	
	**Mean**	**Standard Deviation**	**Mean**	**Standard Deviation**
age	43.96	14.92	41.98	16.9
BMI	23.57	4.54	24.29	4.5
	**TEMPS-I**			
	**<0.5**		**0.5–0.7**	
	**Mean**	**Standard Deviation**	**Mean**	**Standard Deviation**
age	43.27	15.83	42.63	13.84
BMI	23.81	4.52	24.11	4.73
	**TEMPS-A**			
	**<0.5**		**0.5–0.7**	
	**Mean**	**Standard Deviation**	**Mean**	**Standard Deviation**
age	42.24	16.28	45.19	14.28
BMI	23.85	4.76	23.81	4.06

*p* > 0.5 for age and BMI.
